# A potential relationship between soccer shoes and pes cavus: a pilot study

**DOI:** 10.1186/s13102-025-01242-y

**Published:** 2025-07-08

**Authors:** Stephan Becker, Lukas Maurer, Carlo Dindorf, Jonas Dully, Michael Fröhlich, Oliver Ludwig

**Affiliations:** Department of Sports Science, RPTU University Kaiserslautern-Landau, Kaiserslautern, Germany

**Keywords:** Hollow foot, Foot posture, Prevention, Toe gap, Soccer cleat, Arch index, Normalised truncated navicular height

## Abstract

**Background:**

Preventive team testing showed an above-average number of pes cavus among soccer players. This raised the question of whether wearing relatively small soccer shoes - as it is often the case for soccer players - shoes might be responsible and cause a kind of foot compression and a muscular-induced pes cavus.

**Methods:**

This cross-sectional study included 198 male youth and adult soccer players (age: 18.6±5.8 years, height: 176.3±14.8 cm, body mass: 69.9±11.5 kg). The delta between shoe and foot length was compared with arch index (AI) and normalised truncated navicular height (NTNH) and especially for those whose shoes were too small.

**Results:**

The data confirms that among soccer players, 34% had a pes cavus, only 15% were free of pes planus or pes cavus and 27% wore shoes that were too small. Contrary to the authors hypothesis, the NTNH revealed a general relationship with the delta between foot length and shoe length. Players with shoes that were too small had a significantly higher arch (AI = 0.24) than players with adequate shoe size (AI = 0.25), but on average, the arch was still in the range of a normal foot. No correlation was found between the magnitude of the delta and the AI value for the subgroup of players whose shoes that were too small.

**Conclusions:**

The data confirm the increased prevalence of pes cavus among soccer players, but could not confirm the authors’ hypothesis that this may be caused by wearing shoes that are too small.

## Introduction

**Fig. 1:**
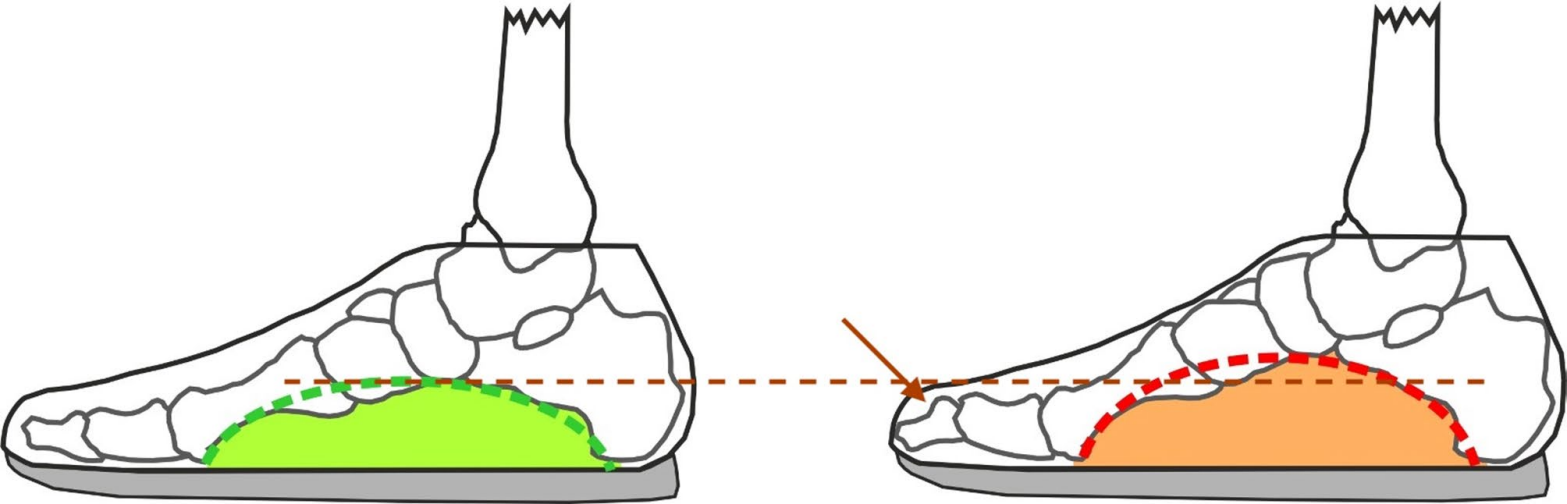
An illustration of the hypothetical development of a pes cavus. On the left, in green, is a foot with adequate shoe size. On the right, in red, the same foot shows a compensatory pes cavus position due to the shoe being relatively small. The longitudinal arch is higher, and the toes are in a flexed position

There are over than 260 million active soccer players worldwide [1], but are they making the right decisions when it comes to choosing the shoe size? The present research question and hypothesis arose during several performance and health diagnostic sessions at the start of a soccer season. The hypothesis is that a too small soccer shoe size might be responsible for the developments of pes cavus in soccer players. The small shoe might be responsible for foot compression and a muscular-induced pes cavus (Fig. 1). By frequently adopting this foot posture during training and matches, it is conceivable that the foot posture could become chronic and promote the development of pes cavus. Research has investigated the change in muscular activation patterns caused by wearing different shoes and supports the thesis that it potentially leads to anatomical and functional changes [2]. Comparable to the conspicuous prevalence observed by Menard et al. (2023), there were a number of analyses of regional youth teams with an increased prevalence of pes cavus diagnoses by the physician, therapist or examiner. In an investigation by Menard et al. (2023) to analyze the relationship between pes cavus and sport injuries, 32% of the soccer players had pes cavus, which is conspicuous compared to the social average [3–5]. It is known that soccer players like to wear tight and small shoes [6, 7], as this provides more support and grip during acyclic changes of direction and allows a better feel on the ball [7, 8].

Assuming an average training load of three training sessions weekly, each lasting approximately 90 min and 34 games per season, a player spends around 369 h annually in a soccer shoe and the number increases further with a further professionalisation. When choosing footwear, soccer players seem to place the highest priority on better performance to the detriment of health aspects such as injury prevention [7]. This may explain the relatively tight and small choice when buying soccer shoes.

A pes cavus is characterised by a raised longitudinal arch and is usually genetically predisposed or develops due to a muscular imbalance in the foot muscles [9]. The pes cavus has a relatively rigid characteristic and the cushioning and shock absorption mechanism, for which the longitudinal arch is partly responsible, is reduced [10]. This can cause a series of overload injuries [11]. Furthermore, compared to a normal foot, the pes cavus has a smaller contact surface on the ground so that the body weight is distributed over a smaller area [10]. The consequence of the reduced contact surface can be higher force peaks and a tendency towards increased foot instability of the foot [3]. As a result, a pes cavus tends to be more susceptible to supination or pronation trauma than a normal foot, and this can be a risk factor for athletes such as soccer players due to the sport’s acyclical changes of direction [12, 13].

During adolescence, the bone and ligament structures of the foot are in a growth process, which is influenced by applied compressive forces [14]. Therefore, these growth phases are particularly susceptible to external disturbances and it could be assumed that the regular constriction of the forefoot in soccer shoes could favor deformities. On the one hand, the growth of the feet increases linearly until with age of 15 [15] and ends at the latest at the age of 17 [16], so there is still a developmental phase before. Older players, on the other hand, have generally already spent more time in their soccer shoes. For this reason, it is important for an initial study such as this one to include a broad spectrum of players to get a descriptive insight.

The aim of this pilot study is to examine a representative sample of soccer players using primarily the arch index (AI) and additionally the normalised truncated navicular height (NTNH). AI would provide information on whether there is an increased prevalence of pes cavus among soccer players. NTNH evaluates the longitudinal arch of the foot and would serve as a further indicator. In addition, a descriptive overview of foot types and foot-to-shoe size ratios is presented. Finally, the data set will be used to compare the measurement methods (AI and NTNH).

1. Aim: Descriptive overview of foot types among soccer players using AI.
2. Aim: Is there a relationship between the delta of foot and shoe lengths and the AI?
*General approach to investigate if there is a relationship between delta values in general and AI.*
3. Aim: Is there a relationship between the delta of foot and shoe lengths and the NTNH in general?
  *General approach to investigate*,* if there is a relationship between delta values in general and NTNH.*
4. Aim: Is there a difference between the subgroups of feet with shoes that are too small (foot length ≥ shoe length) and of those feet with shoes that are bigger than the foot length (foot length > shoe length) as measured by AI?
*Specific pes cavus approach to investigate if players wearing shoes that are too small have a lower AI (pes cavus: AI ≤ 0.21).*
5. Aim: Is there a relationship between the subgroup (shoes too small) and the AI?
*Specific pes cavus approach to investigate if players wearing shoes that are too small have a lower AI (pes cavus: AI ≤ 0.21).*
6. Aim: Are the NTNH and AI measurement methods comparable?

At the time of publication, no comparable studies were available to the authors. To the author’s knowledge, there is only one study on pes cavus and soccer shoes, analysing its prevalence in soccer players and its relationship to foot and ankle injuries [3]. If the suspicion and research question prove to be true, this would possibly be helpful information for the shoe industry and players could be informed about potential side effects of soccer shoes.

## Methods

The article was prepared in accordance with the STROBE guidelines for reporting observational studies [17].

### Participants

This study included 198 male youth players from a German soccer association youth academy and active German adult soccer players (age: 18.6 ± 5.8 years, height: 176.3 ± 14.8 cm, body mass: 69.9 ± 11.5 kg). Since this is a pilot study, no sample size calculation was conducted a priori. All participants were active soccer players between 12 and 37 years old with at least 13.3 ± 5.2 years of soccer experience (minimum: 3 × 90 min practice/week + 1 × 90 min game/week = 6 h/week). Of the 198 players, 114 were youth (< 18 years) and 84 players were aged ≥ 18 years. The participants started playing soccer at the age of 5.6 ± 3.2 years and were active soccer players for 70.3 ± 12.4% of their life time. The exclusion criteria were acute complaints or injuries that could have affected the assessment. This study was conducted in accordance with the current guidelines of the Declaration of Helsinki. It was approved by the responsible ethics commission (No. 55). All participants signed informed consent forms, declaration of participation and provided permission to publish their results. Participants aged < 18 years had their legal guardians sign the documents.

### Experimental design

In this study, a cross-sectional design to analyse possible effects of the size of soccer shoes on foot posture was used. Each participant attended one data-collection session.

### Experimental procedure

**Fig. 2:**
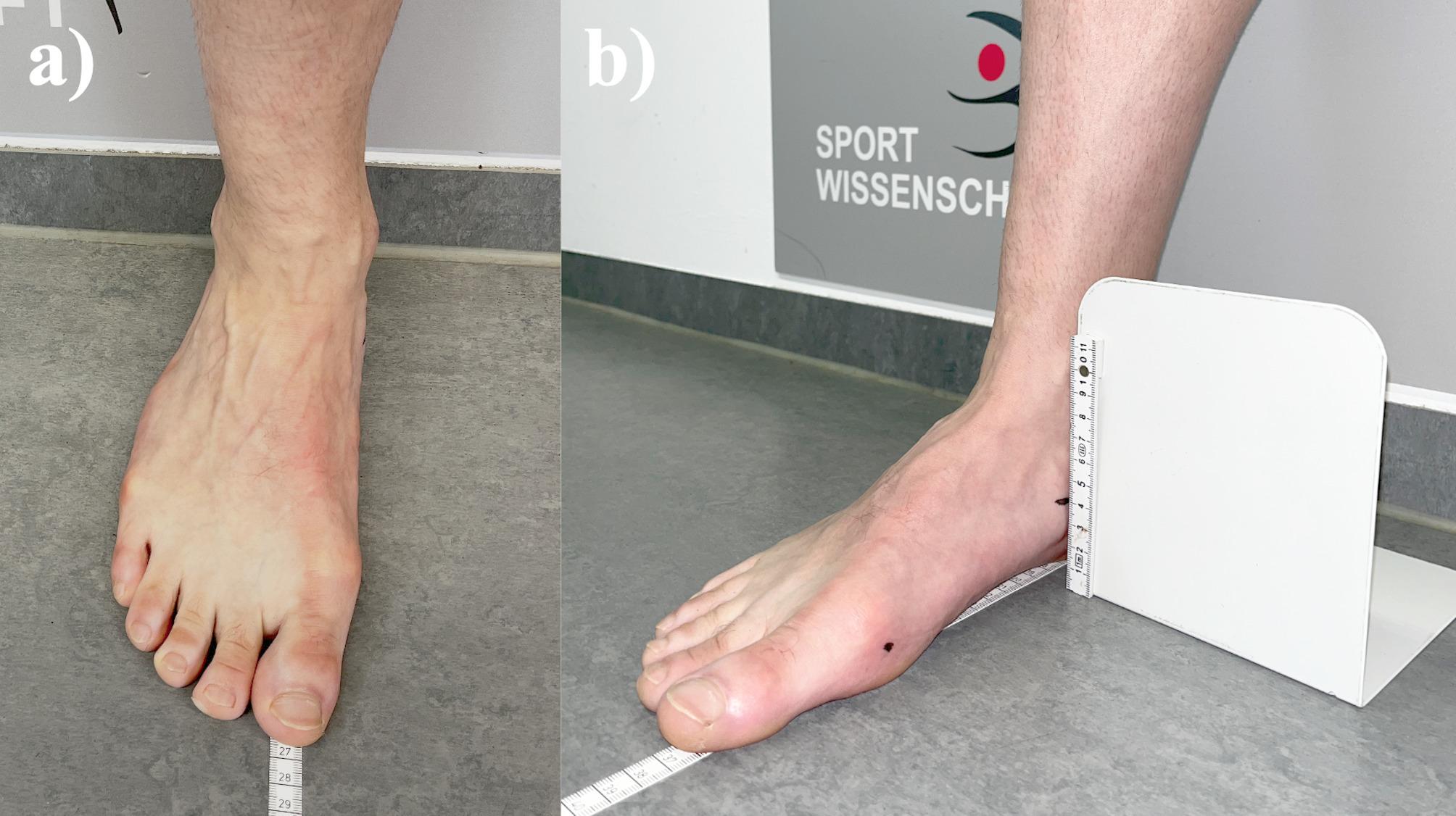
Assessment of (**a**) the foot length (**b**) the navicular height

**Fig. 3:**
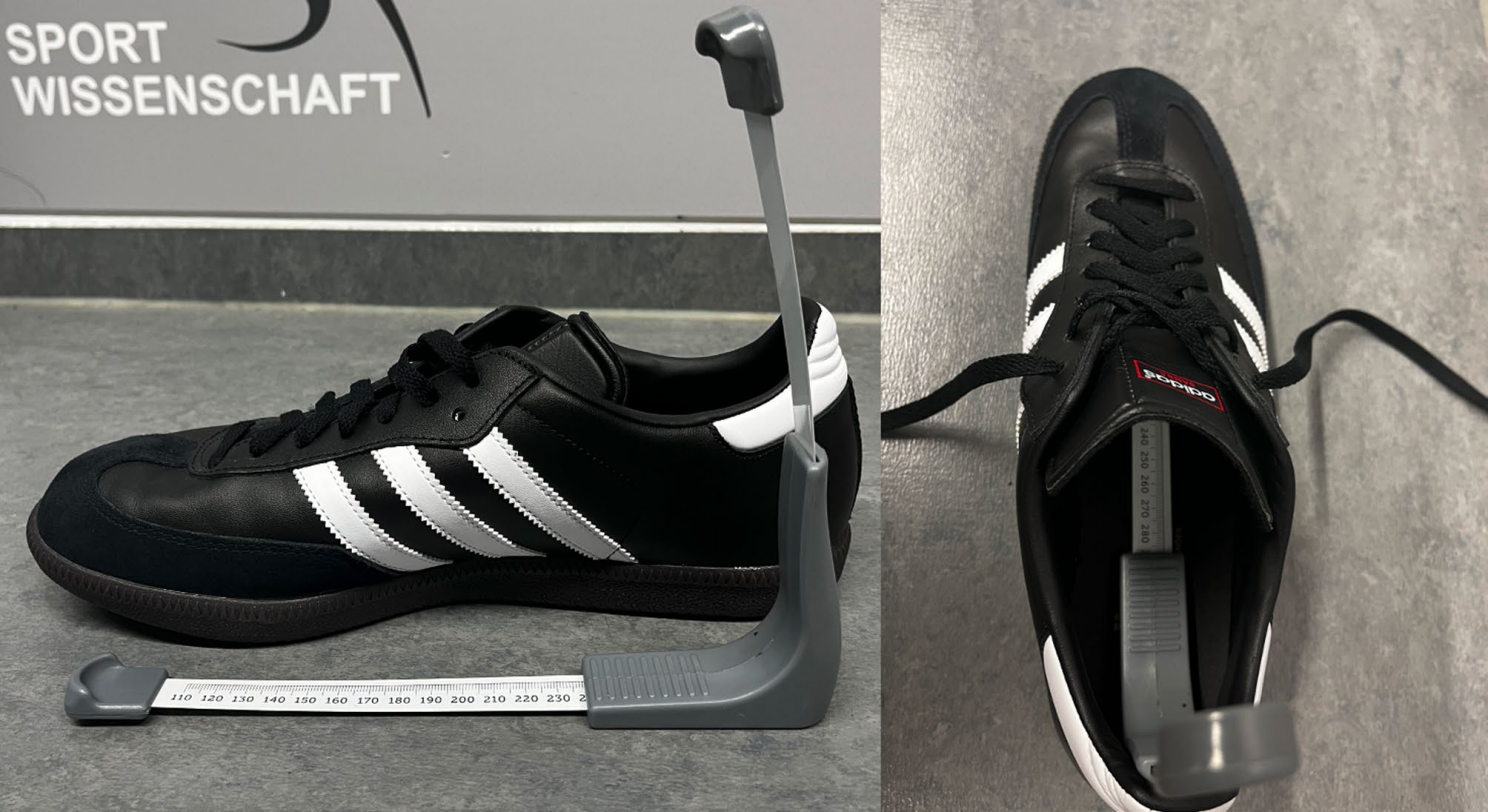
Assessment of shoe length

The participants arrived rested before practice to avoid bias as a decreasing of the longitudinal arch due to fatigue [18]. All measurements were conducted in a separate room of the respective sports club (preseason 2023 and 2024) by an experienced researcher in this field. The participants received standardised instructions on the study and its procedure. Using a standardised measuring tape, the length (in cm) of both feet was measured in a standing position from the most prominent and distal part of the calcaneus to the most distal part of the longest toe (Fig. 2), which usually is the first or second toe. A right-angled slider (Fig. 2) was utilised to ensure maximum measuring accuracy for the assessment of NTNH (see 2.5.). Afterwards, the inner shoe size was measured with an inner shoe measuring device (Fig. 3; ZentiMetrix^→^, Berg GmbH & Co KG, Trier, Germany). These two values were used to determine the delta between foot and shoe length (delta = shoe length– foot length), with zero and negative values indicating that a shoe is too small (too small = foot length ≥ shoe length). Throughout the next steps the data required to determine the AI (see 2.4) and NTNH (see 2.5) were captured.

### Arch index (AI)

**Fig. 4:**
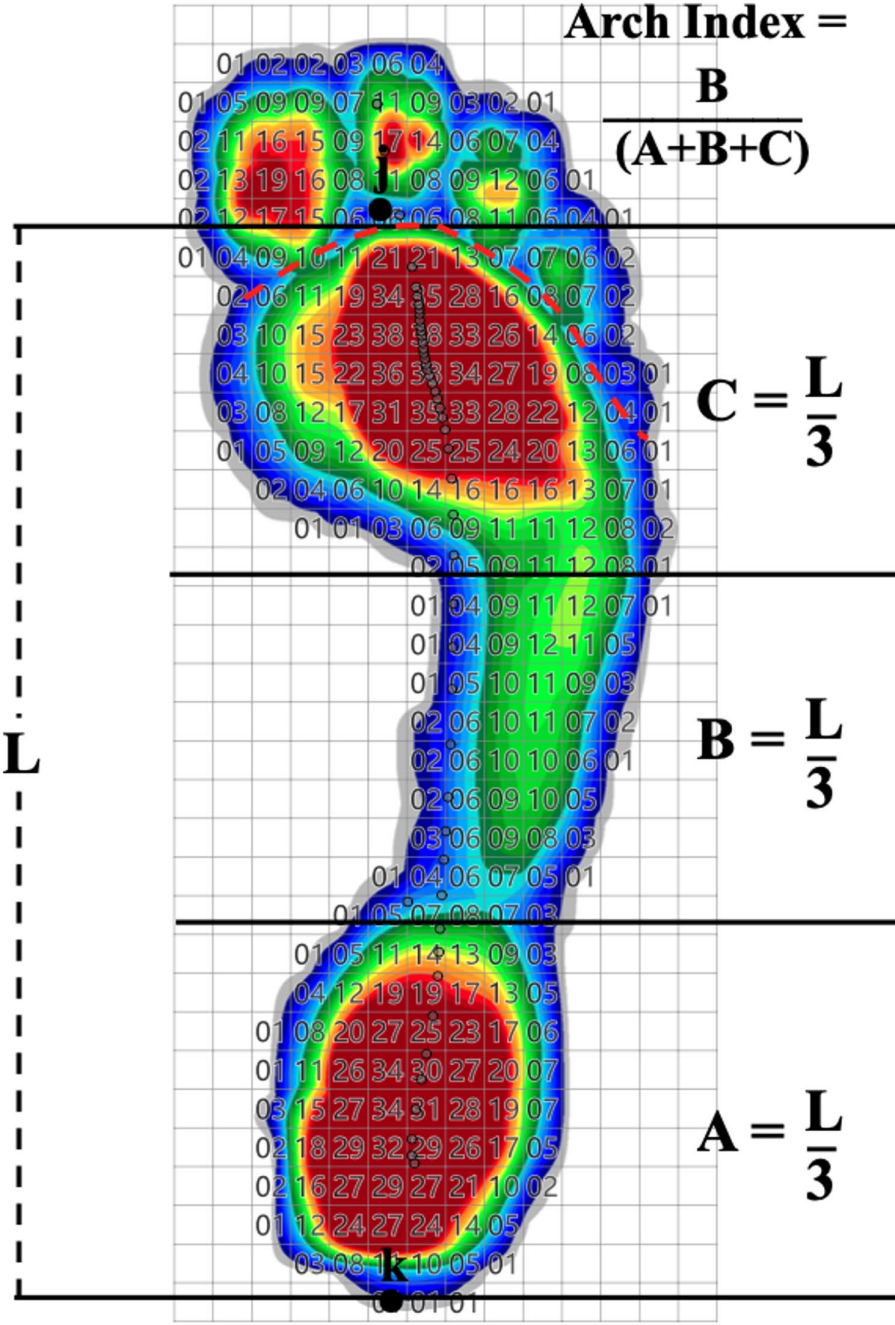
Assessment of the Arch Index (AI)(L = foot length)

The AI helps to rate the longitudinal arch of the foot [19, 20]. The AI is calculated based on a digital footprint during standing or walking (Fig. 4). The footprint was taken using a pressure measuring plate (60 × 40 cm; Zebris Medical GmbH, Isny, Germany). The foot length (L) represents the distance between the most dorsal part of the calcaneus (k) and to the most ventral part of the forefoot metatarsal (j). The toe marks play no role in determining the AI. The foot length (L) is then divided into three equal segments (A, B, C) so that AI corresponds to the quotient of B by the sum of all segments (A + B + C). The AI enables the rating of the longitudinal foot arch: normal foot (0.21 < AI < 0.25), pes cavus (AI ≤ 0.21) and pes planus (AI ≥ 0.26) [21].

For the measurement, the players walked over the pressure measuring plate embedded in the floor with a run-up of at least three steps to ensure a gait pattern that was as natural as possible. The program then generated the pedographic pressure distribution of the foot so that the AI could be calculated.

### Normalised truncated navicular height (NTNH)

**Fig. 5:**
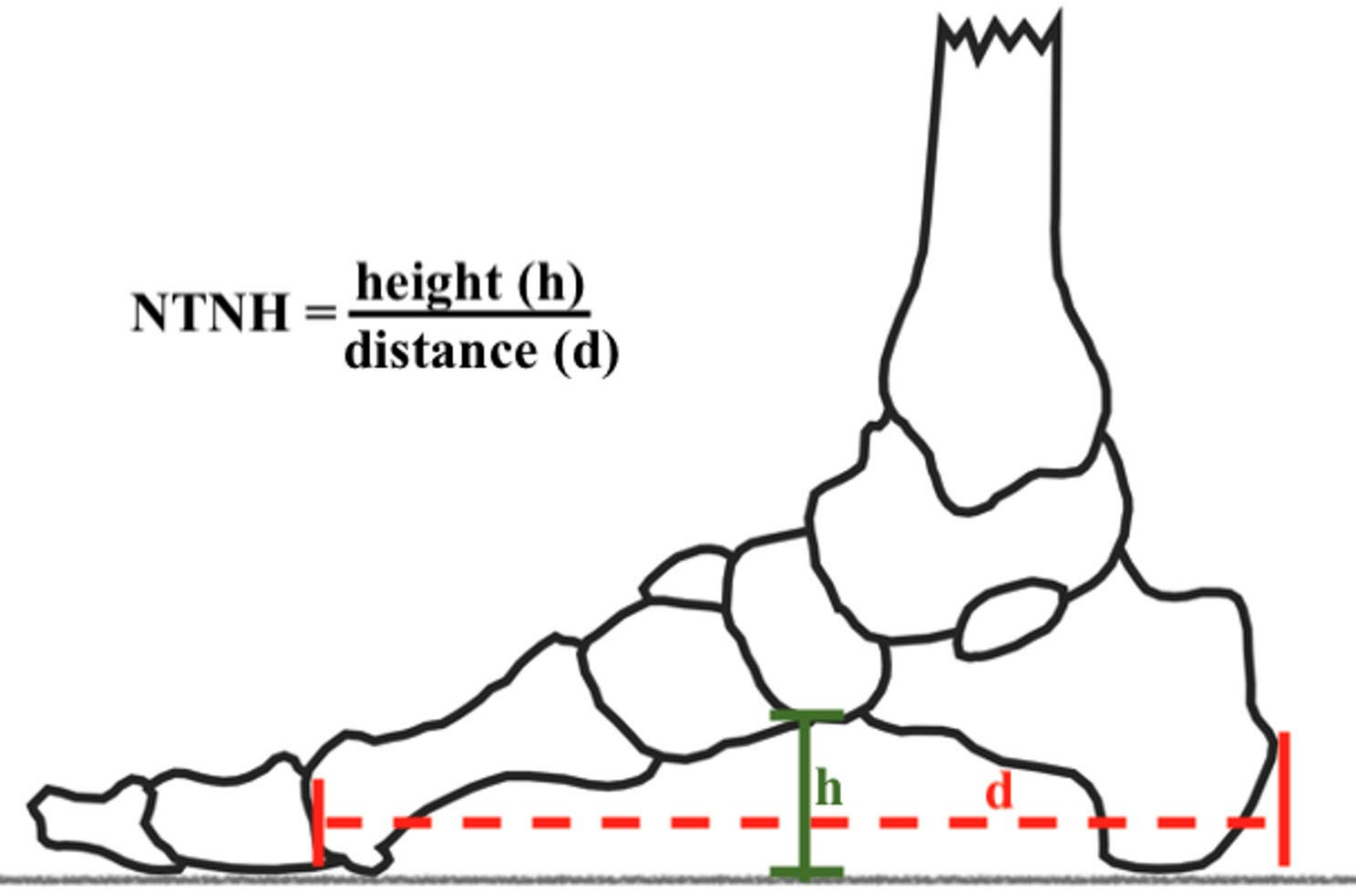
Assessment of the normalised truncated navicular height (NTNH)

The NTNH is a measurement that originates from radiology, where the method is called navicular index and is used to assess the longitudinal arch of the foot [22, 23]. For this purpose, the truncated length of the foot is set in relation to the height of the arch (Fig. 5). The truncated length corresponds to the distance (d) between the most dorsal part of the calcaneus to the centre of the metatarsophalangeal joint. The height (h) is the distance between the floor and the anterior-inferior part of the os naviculare (Figs. 3 and 5). All anatomical landmarks can be easily palpated, with proofed reliability, validity and diagnostic accuracy [23]. A foot with NTNH ≤ 0.195 is considered as a flat foot. Since there is still no NTNH cut-off point for pes cavus, this diagnostic cut-off is not directly necessary, seeing that it can be classified by the AI. Still, the higher the NTNH value, the higher the longitudinal arch in relation to the truncated length.

The navicular and first metatarsophalangeal joint were first marked for the measurement. The players then positioned themselves against the wall so that the calcaneus was in contact with the wall and the truncated foot length (d) and the height of the longitudinal arch (h) could be measured.

### Statistical analysis

For research questions 2–6, the statistics were calculated in R (version 4.4.2). Homoscedasticity was tested using the Levene test, and normal distribution was tested using Shapiro-Wilk test and Q-Q-plots. For research questions 2 and 3 mixed general model was calculated using the lme4 package. One factor was the foot side (left or right), and another factor was the delta between the shoe length and foot length. The dependent variables were the AI for research question 2 and the NTNH for research question 3. For both, a zero model was calculated, that is without the fixed effects (delta and foot side). In addition, a new model was calculated for both, first with the delta and a second model including the side. Intraclass-correlation (ICC), Akaike information criteria, and Bayes information criteria were calculated to evaluate the usefulness of the predictors. For research question 4, an unpaired Wilcoxon rank sum test was calculated to evaluate the differences in the AI between the groups with too small shoes (delta ≤ 0.0 cm) and the group with larger shoes (delta > 0.0 cm). For research question 5, a Spearman correlation was performed between the delta and the AI for the group with too-small shoes (delta ≤ 0.0 cm). Regarding research question 6, a linear regression was performed using the lme4 package as well.

## Results

The following three sections present the results for the various research questions and research aims 1–6.

### Distribution of foot types among soccer players (1st aim)

**Fig. 6:**
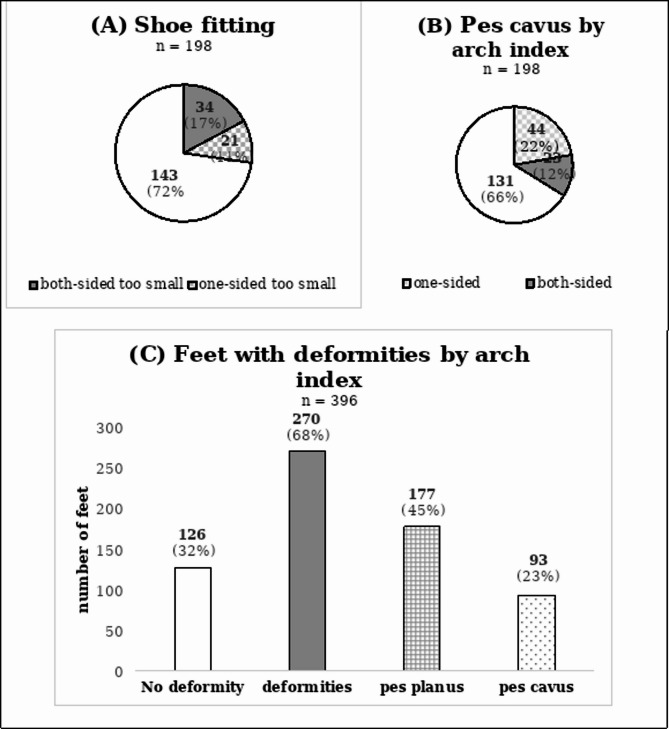
Overview of (**A**) shoe fitting of all players (*n* = 198); (**B**) number of pes cavus diagnosed by the arch index (AI) (*n* = 198) and (**C**) number of feet (*n* = 396) with no deformities, deformities, pes planus, and pes cavus measured by the AI

Table 1; Fig. 6 provide an overview of the key descriptive values that may be significant for data interpretation. Of the198 players, 29 players (15%) showed no foot deformity, while 169 players (85%) showed at least one deformity (pes cavus, pes planus) measured by the AI. An extended, descriptive overview of age-related differences can be found in Table 2.

**Table 1:** Descriptive data of the sample (*n* = 198)

Variable	Mean ± SD	Minimum	Maximum
Shoe length [cm]	27.1 ± 1.3	23.6	30.3
Foot size: right side [cm]	26.4 ± 1.3	23.1	29.3
Foot size: left side [cm]	26.4 ± 1.3	23.0	29.8
Δ shoe-foot: right side [cm]	0.7 ± 0.8	-1.3	3.6
Δ shoe-foot: left side [cm]	0.7 ± 0.8	-1.7	3.0
AI: right side [cm]	0.25 ± 0.06	0.06	0.48
AI: left side [cm]	0.24 ± 0.05	0.09	0.36
NTNH: right side [cm]	0.21 ± 0.04	0.12	0.32
NTNH: left side [cm]	0.21 ± 0.03	0.14	0.31

**Table 2:** Descriptive age-related data of the sample (*n* = 198)

	Players < 18 years Total: 114 players	Players ≥ 18 years Total: 84 players
Pes cavus	38 (33%)	29 (35%)
Pes cavus & shoes too small	11 (10%)	10 (12%)
Pes planus	65 (57%)	44 (52%)
Pes planus & shoes too small	15 (13%)	5 (6%)

### Relationship between tight soccer shoes and the development of pes cavus (2nd, 3rd, 4th and 5th aim)

The final mixed linear model for AI (2nd aim) showed the following characteristics for the delta between shoe and foot lengths, as well as the side of the foot (Table 3).

**Table 3:** Mixed linear model for delta foot and shoe length vs. arch index (AI)

Variable	Estimate	Std. Error	df	t-value	*p*
Intercept	0.248	0.007	362.5	36.490	< 0.001**
Delta	0.002	0.004	313.1	0.497	0.620
Side	-0.004	0.004	196.5	-1.070	0.787

Since none of the predictors showed significant results, the evaluation criteria for the first and second models are not presented.

The final mixed linear model for NTNH (3rd aim) shows the following characteristics for the delta between shoe and foot lengths, as well as the side of the foot (Table 4).

**Table 4:** Mixed linear model for delta foot and shoe length vs. normalised truncated navicular height (NTNH)

Variable	Estimate	Std. Error	df	t-value	*p*
Intercept	0.207	0.004	387.5	54.115	< 0.001**
Delta	0.009	0.002	358.9	4.045	< 0.001**
Side	-0.001	0.001	181.6	-0.271	0.787

It can be seen that the delta has a significant effect on the NTNH. The larger the delta between shoe and foot lengths, the bigger the NTNH value.

The median AI of the subgroup of feet with too small shoes (89 feet, delta ≤ 0.0 cm) was 0.24 (interquartile range (IQR) = 0.044) and with larger shoes (*n* = 307, delta > 0.0 cm) 0.25 (IQR = 0.063). Wilcoxon rank sum test showed a significant difference with a small effect size (*p* = 0.0495; *r* = 0.099; CI = [0.01−0.18]) (4th aim).

Spearman correlation showed no effect between the delta and the AI within the subgroup of players wearing shoes that are too small (*r* = 0.001, *p* = 0.99) (5th aim).

### Comparison of normalised truncated navicular height (NTNH) and arch index (AI) (6th aim)

The linear model comparing the two methods in focus can be described with the following formula:

$$\:AI=0.329-0.401*\text{N}\text{I}$$

**Fig. 7:**
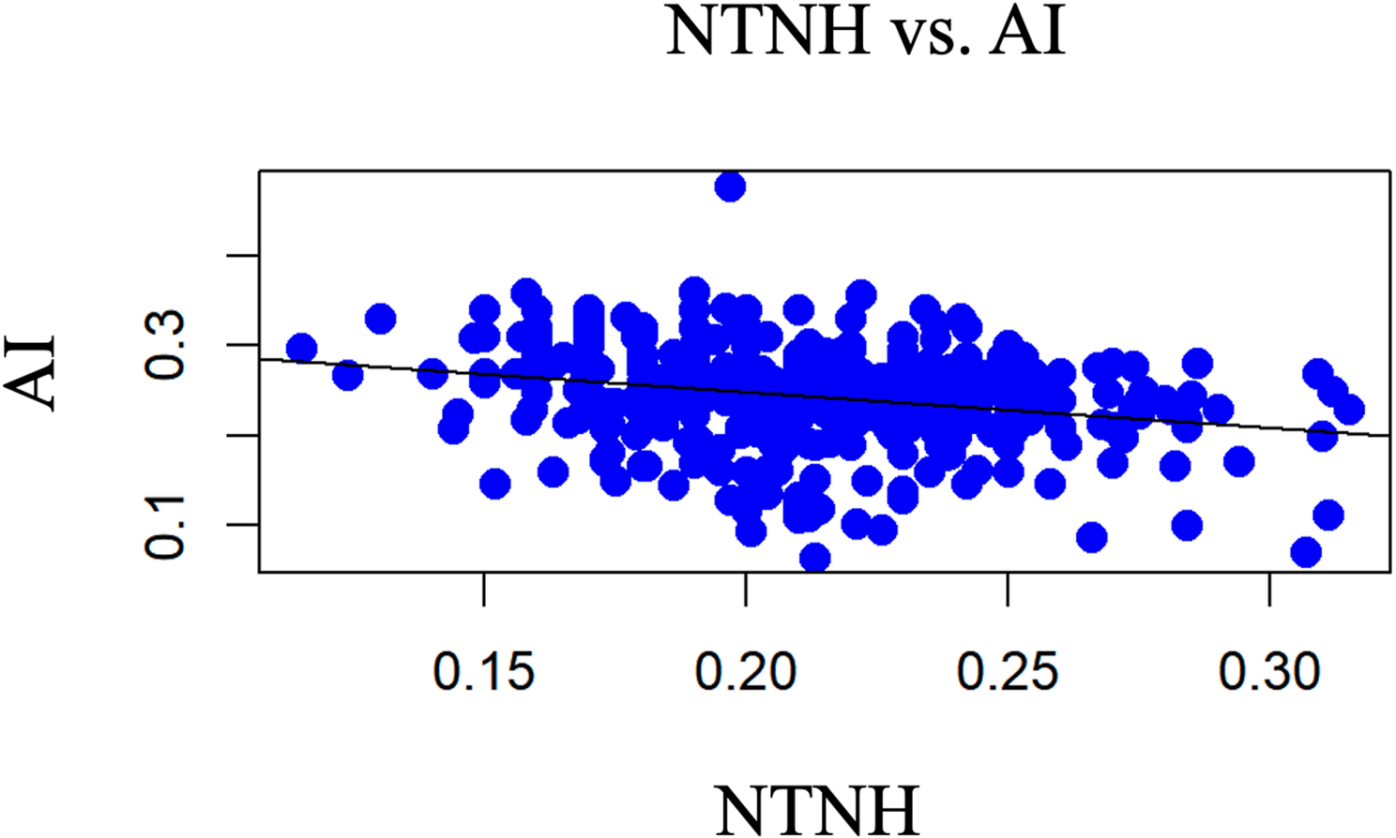
Comparison of normalised truncated navicular height (NTNH) and arch index (AI)

This regression was statistically significant with a small effect (adjusted R² = 0.062, F(1, 394) = 27.18, *p* < 0.001**)(Fig. 7).

## Discussion

The descriptive data showed that there were 67 players with at least one pes cavus and 90 feet with pes cavus in total. The combination of pes cavus and shoes that are too small applies to 11% of the sample (21 out of 198 players). In addition, 54 players wore shoes that were too small, independent of the foot shape (1st aim). No effect was observed for the delta and the AI, indicating that no relationship was observed between the delta of shoe and foot lengths and AI (2nd aim). Contrary to this assumption, a significant relationship could be observed for NTNH and the delta between the shoe und foot lengths (3rd aim). A significant difference was found for the AI between players wearing shoes that are too small (AI = 0.24) and those wearing shoes that are not too small (AI = 0.25) (4th aim). No relationship was found between the AI and the delta of players within the subgroup of players whose shoes were too small (5th aim). Furthermore, only a minor relationship was observed between the two methods of measurement. The AI decreased with an increasing NTNH. Reducing the NTNH by 0.1 results in an increase of 0.04 in the AI (6th aim).

### Distribution of foot types among soccer players (1st aim)

The descriptive data show that out of the 198 players examined, 67 players (34%) had a pes cavus on at least one side. If one adheres to the hypothesis that the shoe size is the cause of the formation of a pes cavus, then the question arises as to why this only occurs unilaterally in some parts? It could be due to a different requirement profile between the stance leg and the kicking leg [24] or to a difference in foot lengths with symmetrical shoe sizing [25]. As this is the first study in this direction, this would have to be evaluated in further studies.

Menard et al. (2023) analysed the relationship between the cavus foot type, and foot and ankle injuries of soccer players. Of the 277 players, 88 players (32%) had a pes cavus, which is almost identical to our results and higher than in other ethnic populations [3–5]. If we summarise all deformities, then a pes cavus or a pes planus was diagnosed in 167 out of 396 feet (42%), and 169 out of 198 players showed at least one deformity (pes cavus, pes planus) which equals about 85% of the population. From the authors’ point of view, those are serious numbers, knowing that foot deformities are also counted among the intrinsic risk factors for overuse and traumatic injuries [26, 27]. The results show that it is worthwhile for soccer players and professional clubs to examine players’ feet as part of preventive diagnostics [3, 18] to be able to intervene in foot orthoses, if necessary [28]. Even if the study situation on the targeted effectiveness of foot orthoses is not always clear, it seems logical that the kinematics and kinetics of the foot can be changed with supportive elements [28, 29].

In addition, 54 players (27%) wore shoes that tended to be too small (Δ shoe-foot: 0.0 cm to -1.7 cm). This is worth a discussion since there is no consensus on the proper shoe size with practical recommendations. The size depends on personal preferences and experiences [30]. Running and athletic shoes for instance typically should have some distance between the longest toe and the end of the shoe [31, 32], while soccer shoes require a tighter fit [33]. In addition, the timing of the examination also plays a role, especially for players who are still growing up. Young people and their parents probably tend to buy shoes half a size too big, knowing that their feet are still growing. There is no clear indication of how many millimeters or centimeters larger soccer shoes should be than the foot [31, 32]. This allows the foot to have enough space in the shoe to avoid discomfort and deformities and prevent the toes and toes nails from hitting the shoe [34, 35], or to allow room for expansion depending on the time of day [36]. Therefore, the authors defined too small = shoe length ≤ foot length.

Overall, there are relatively many pes cavus among soccer players, but only 11% of the players with pes cavus also wore shoes that were too small.

### Relationship between soccer shoes and the development of pes cavus (2nd, 3rd, 4th and 5th aim)

The main aim of this study was to analyse whether wearing soccer shoes might be responsible for a kind of foot compression and a muscular induced pes cavus. The first step was to check whether there was a general correlation between the delta and the AI or NTNH. The aim was to check whether the wearing habits of the shoe, in the sense of the delta between the shoe and foot lengths, reveal a co connection to the foot type (normal foot, pes planus, pes cavus). There was no correlation between the delta of foot and shoe lengths and the AI. However, there is a significant effect of the NTNH but the effect is contrary to the authors’ hypothesis. Based on this sample, the NTNH value increases as the delta increases. A larger NTNH value corresponds to an increase in arch height. Consequently, the result means that the greater the delta between the foot and shoe lengths, the higher the arch height of the player.

Since this is a pilot study and the topic was to be investigated in general terms for the first time, the focus was on possible general correlations (2nd, 3rd aim) and not just on players with pes cavus or shoes that are too small (4th, 5th aim). Nevertheless, the reason for this correlation in the delta and NTNH must be questioned (3rd aim). One conceivable explanation is that players with a pes cavus typically have a high longitudinal arch, and therefore, tend to buy a slightly larger shoe so that there is room for the high arch and ensure that the shoe is comfortable to wear. In turn, this increases the delta. Consequently, it was apparent that players with a pes cavus tend to intentionally or unintentionally choose a larger size when buying their shoes. However, an examination of the descriptive data of players with pes cavus revealed no systematic patterns.

For a more specific insight, players with shoes that were too small were first identified, and their AI values were compared with those of players with adequate shoe sizes (4th aim). On average, the AI values of the players with shoes that were too small were significantly lower on average (AI = 0.24) than that of other players (AI = 0.25). However, pes cavus is represented by an AI of ≤ 0.21. Thus, the present results cannot be seen as a confirmation of the research question, since the AI valus of 0.24 is classified as normal and inconspicuous. The result only supports the hypothesis that within the present sample, players with shoes that were too small had a slightly higher longitudinal arch than players with adequate shoe size.

In the subgroup of players with shoes that were too small, no correlation was found between the AI values and delta (5th aim). In general, there seem to be a few indications, such as the prevalence of pes cavus among soccer players. However, the pilot study did not allow a more precise interpretation of the research questions. Future studies should continue to support the overview of the prevalence of pes cavus and soccer players [10, 3]. Further research questions with a similar hypothesis should be limited to adult professional soccer players (four practises/week + match) who have been playing soccer intensively since early life. It can be assumed that they have spent more time wearing soccer shoes than semi-professionals [37]. Furthermore, large samples sizes are needed to obtain the cut-off values for NTNH and pes cavus, as is the case for AI. In this study the 90 feet with pes cavus had an average NTNH of 0.17 ± 0.04.

### Comparison of both measurement methods (NTNH, AI) (6th aim)

The longitudinal arch of the foot is important in many diagnoses, as a deviation can be the cause of a number of injuries [38]. Evaluating the foot type based solely on the visual impression of the examiner is inconsistent even among experts [39]. Consequently, a number of methods that focus on evaluating longitudinal arch structures have been. Footprint-based parameters include the Steheli index, Chipaux-Smirak index, truncated arch index, arch index, arch length index, arch angle, truncated arch index and modified arch, and footprint indices [40]. Dimension-related indices included the navicular index, arch height index, normalised navicular height, navicular drop, navicular drift [40]. These techniques use radiographs, ultrasound, footprints, bony landmark measurements and clinical assessments [41]. The data collected in this study should be used secondarily to compare the methods NTNH and AI methods. This regression was statistically significant with a small effect. An increase of the NTNH by 0.1 results in a decrease of 0.04 in the AI. As a reminder, pes cavus is present, if the AI is ≤ 0.21 and the NTNH value increases with the arch height. The NTNH was obtained from the relationship between the longitudinal arch height (os naviculare) and truncated foot length. NTNH effectively differentiates between flatfoot and normal-arched feet with high sensitivity and specificity [22]. AI evaluates the longitudinal arch based on the plantar pressure area and moderate to high correlation with most foot-type metrics and can predict standing arch heights using regression analysis [42, 40].

The results of this study are consistent with those of Jonely et al. (2011) who compared the relationships between the clinical measures of static foot posture and plantar pressure during static standing and walking [43]. They were able to demonstrate a significant correlation between different pressure zones (hallux, medial forefoot, and medial midfoot) and the clinical test procedures (arch index, navicular drop, and navicular drift). However the strength of these relationships ranged from poor to fair. Similar results were reported by the study of Murley et al. (2009), which aimed to compare different clinical and radiographic measurements to classify foot posture [44]. They compared NTNH with AI and reported a relationship with poor to fair strength.

Newer methods, such as three-dimensional movement analysis of the rear- and midfoot offer the best ways to analyse the foot [45]. However, NTNH and AI, for example, are still widely used because they are quick, easy and cost-effective [23]. These measurements provide researchers and clinicians with tools to assess foot arch characteristics and their potential implications for injury prevention and treatment [40]. NTNH [23] and AI [19, 20] are reliable and valid methods, with obviously different strengths and weaknesses. NTNH is efficient, and has low acquisition costs, and has good anatomical landmarks. AI is more objective but requires the acquisition of a force plate, whereby soft tissue can cause bias [46]. Both procedures appear to be negatively influenced by valgization or eversion of the calcaneus. In addition, it should be noted that the majority of studies have compared different methods to classify normal feet and pes planus [47, 22, 23], but not the classification for pes cavus. Therefore, a well-considered decision is required when choosing the right method depending on the research question, which is probably why such a large number of existing measurements for the longitudinal arch have been developed.

## Limitations

This pilot study has several limitations that must be considered when answering the research question and continuing research. First, it should be emphasised that this is a pilot study with a cross-sectional design on a practical question. Accordingly, further research methods are needed to answer the possible connection between soccer shoes and their potentially negative influence on foot posture. Because of the pilot study character no sample size calculation a priori was applied. The study sample did not include any female players. Hence, we cannot make any statements about the effects of the soccer shoe on female gender. Furthermore, young players were also included in the study sample. Based on the hypothesis and research question, it can be assumed that a longer wearing time of the soccer shoes in an absolute and proportional sense should actually increase the effect of the shoe on the feet of players. The definition of whether a shoe is too small is debatable and depends on the individuality of the player and the specific characteristics of the shoe model. However, even with this tolerant definition of too small, effects were found. Finally, it should always be considered that the delta between shoe and foot lengths only represents a moment in time, especially when conducting research on young people. Nonetheless, in this study, the aim was to investigate whether there was an observable tendency. In addition, the player’s feet may still be growing, a larger shoe size may have been bought deliberately, or the feet may be fully grown but different models turn out differently, so that the snapshot may not correspond to the predominant condition. Finally, it should be considered whether other shoe factors are related to the hypothesis (forefoot and rearfoot width), as foot length alone may not be sufficient to judge the shoe size [48].

Future studies are recommended to favour longitudinal studies and samples with only players who wear shoes that are too small.

## Conclusion

The descriptive data show that there were 67 players (34%) with at least one pes cavus. In addition, 27% of the players wore shoes that had the exact length of the shoe or smaller (*n* = 54) but only 11% of the players with pes cavus (AI) also wore shoes that were too small. Still, players with shoes that were too small had an higher arch (AI) than players with adequate shoe size. No correlation was found between the magnitude of the delta and the AI value.

The pilot project shows that there is an increased number of pes cavus in male soccer players. The potential causes of this and, in particular, the effect of soccer shoes on the development of foot deformities in general remains an important perspective and needs to be investigated further. This way, the shoe industry could be helped and players could be educated and sensitized.

## Data Availability

The study data are available from the corresponding author upon reasonable request.

## Abbreviations

AI: Arch index
NTNH: Navicular truncated navicular height
